# Interepidemic xenosurveillance of Japanese encephalitis virus and Zika virus in *Culex* mosquitoes from Ubon Ratchathani province, Thailand

**DOI:** 10.14202/vetworld.2024.1555-1561

**Published:** 2024-07-21

**Authors:** Wilasinee Surasa, Chamsai Pientong, Tipaya Ekalaksananan, Hans Jorgen Overgaard, Sirinart Aromseree, Supranee Phanthanawiboon

**Affiliations:** 1 Department of Microbiology, Faculty of Medicine, Khon Kaen University, Khon Kaen, Thailand; 2 HPV&EBV and Carcinogenesis, Faculty of Medicine, Khon Kaen University, Khon Kaen, Thailand; 3 Department of Medical Entomologist, Faculty of Science and Technology, Norwegian University of Life Sciences, Ås, Norway

**Keywords:** *Culex*, Japanese encephalitis virus, Thailand, xenosurveillance, Zika

## Abstract

**Background and Aim:**

Some *Culex* mosquitoes are competent vectors for Japanese encephalitis virus (JEV) and Zika virus (ZIKV), which cause public health problems worldwide, especially in South-east Asia. Xenosurveillance of *Culex* mosquitoes remains limited compared with other common mosquito-borne diseases. This study aimed to identify JEV and ZIKV in field-caught *Culex* mosquitoes collected from Ubon Ratchathani province.

**Materials and Methods:**

We investigated the presence of JEV and ZIKV in *Culex* mosquitoes from two districts in Ubon Ratchathani province, Thailand, and examined their role in viral interepidemic circulation. Female *Culex* mosquitoes (5,587) were collected using a mechanical aspirator from indoors and outdoors. The consensus sequences of the E and NS1 genes of JEV and the E gene of ZIKV were identified using real-time reverse transcription-polymerase chain reaction.

**Results:**

From 335 sample pools that contain a total of 5587 adult female Culex mosquitoes collected from Don Yung, Mueang district (4,406) and Phon Duan, Det Udom district (1,181), none of the collected mosquitoes tested positive for either JEV or ZIKV.

**Conclusion:**

This study did not find JEV and ZIKV in *Culex* mosquitoes collected from the area of collection, which may be due to the low circulating amount of the virus in the vectors in the area, making it undetectable, or it may be because *Culex* mosquitoes are not suitable vector for the virus being tested. However, further xenosurveillance study of JEV and ZIKV in mosquito is suggested to prepare for the next outbreak.

## Introduction

Japanese encephalitis and Zika fever are caused by the Japanese encephalitis virus (JEV) and Zika virus (ZIKV), respectively, transmitted by mosquitoes. Japanese encephalitis is a zoonotic disease that causes widespread neurological infections in South and South-east Asia and Northern Australia [1]. More than 60,000 neuroinfections occur annually in over 50 endemic countries, mainly in tropical regions, due to Japanese encephalitis. Human cases of JEV infection in Thailand were reported in 1970s and 1980s [2]. JEV immunization, initiated as a part of the childhood vaccination program in 1990, led to a decrease in Japanese encephalitis (JE) cases [3]. Despite efforts to prevent JEV through vaccination, education, and control strategies, cases still emerge in Thailand. Twenty-two cases of JE have been reported in several locations in Bangkok and Hat Yai district, Songkhla province [2]. From 2018 to 2021, the Ubon Ratchathani province had the highest morbidity rate for Japanese encephalitis in Thailand [4]. During the 1980s, JEV genotype 3 (GIII) was the predominant strain, whereas, since 2003, it has been displaced by genotype 1 (GI) [5, 6]. The primary vector of JEV is *Culex* *tritaeniorhynchus*, but *Culex gelidus*, *Culex fuscocephala*, and *Culex quinquefasciatus* can also be potential vectors for JEV transmission [7]. The ZIKV is spreading to many African, Asian, Pacific Island, and American countries [8]. The discovery of ZIKV occurred in Thailand in 1954 [9]. In 2012–2014, the first autochthonous cases of the disease were identified among Thai residents in various provinces [10]. In 43 Thai provinces, 2,300 cases of ZIKV infection were identified between 2016 and 2018 [11]. In 2019, 273 cases of Zika fever were reported by the Bureau of Epidemiology, and two of them were found in Ubon Ratchathani province [12]. While Aedes mosquitoes are the primary transmitters of ZIKV, evidence hints at *Culex* mosquitoes, particularly *Cx. quinquefasciatus*, playing a role as well [13, 14]. The controversy over using the *Culex* mosquito as a vector for ZIKV remains unresolved. The integrative review by Viveiros-Rosa *et al*. [15] revealed that ZIKV can be detected in salivary glands of *Cx. quinquefasciatus*, *Culex restuans*, *Culex tarsalis*, and *Culex coronator*, but there is still a lack of evidence indicating that *Culex* is a competent ZIKV vector.

Xenosurveillance detects pathogens in vertebrate hosts by analyzing blood samples taken from feeding arthropods [16]. In Thailand’s JEV and ZIKV surveillance studies, the utility of xenosurveillance, despite being a non-invasive and straightforward method, is limited. *Culex* mosquitoes’ role in transmitting JEV and ZIKV warrants further investigation. Conducting a xenosurveillance study in *Culex* mosquitoes can reveal the viral condition of both the vectors and their vertebrate hosts.

In north-eastern Thailand’s Ubon Ratchathani province, we used a retrospective molecular investigation to study the co-occurrence of viruses in urban and rural *Culex* mosquitoes. The mosquito distribution was assessed at both research locations. This information aids in understanding the prevalence of JEV and ZIKV in mosquitoes for future epidemiological studies. The findings could contribute to the development of effective vector control strategies against arbovirus diseases in Thailand.

## Materials and Methods

### Ethical approval

Ethical approvals were obtained from the Khon Kaen University Ethics Committee for Human Research (KKUEC, approval numbers HE671215 and HE641166).

### Study period and location

The study was conducted from January 2019 to December 2019. Adult mosquitoes were collected in Don Yung, Mueang district (N15.2926667, E104.8203056) and Phon Duan, Det Udom district (N14.6471944, E105.0929167) in Ubon Ratchathani province.

### Sample collection sites and mosquito collection

These collection sites were part of the DENCLIM project mentioned and are further described in Rahman *et al*. [17]. The sample sites were selected on the basis of population, feasibility, and logistics.

Mosquitoes were collected indoors and outdoors from 90 households using a Prokopack aspirator [18]. The collected samples were shipped to the laboratory at Khon Kaen University by an appropriate cold chain transport company. Female mosquitoes were morphologically identified as species. Ten to 20 female *Culex* mosquitoes were pooled according to the study site and collection area (indoor or outdoor) and then stored at −20°C for subsequent analysis.

### Viral genomic RNA isolation and quantification

The sample pools were homogenized using a micropestle with L-15 medium (Thermo Fisher Scientific, Waltham, USA). After centrifugation, the supernatant of each sample was collected and processed for viral RNA extraction. Viral RNA was isolated using the QIAamp viral RNA mini kit (Qiagen, Hilden, Germany) according to the manufacturer’s instructions. The purity and concentration of the obtained viral RNA were quantified through 260/280 nm absorbance using NanoDrop 2000/2000c spectrophotometers (Thermo Fisher Scientific, USA).

Actin-1, a mosquito housekeeping gene, was detected to qualify the RNA extracted from the samples. Randomized cDNA converted from 86 RNA samples was amplified by real-time polymerase chain reaction (PCR) using universal primers: Act-2F, ATG GTC GGY ATG GGN CAG AAG GAC TC, and Act-8R, GAT TCC ATA CCC AGG AAG GAD GG, following the study of Staley *et al*. [19] for actin-1 detection. Thermocycling conditions were as follows: 95°C for 3 min, followed by 40 cycles of 95°C for 30 s, annealing for 30 s at 55°C, and extension for 30 s at 72°C.

### JEV and ZIKV detection in mosquitoes

The extracted viral RNA samples were converted to complementary DNA (cDNA) using the SuperScript®III first-strand synthesis system (Thermo Fisher Scientific, USA). This process was facilitated by specific primers: JEV Eb-Rv, GTGTCRGCATGCACATTGGTMGC, or ZIKV-Rv, CACCARRCTCCCYTTGCCA. Subsequently, real-time reverse-transcribed PCR (RT-PCR) was employed to amplify regions of interest within viral cDNA samples, specifically targeting the E and partial NS1 gene of JEV or the E region of ZIKV. The specific primers for the detected E and partial NS1 genes of JEV were designed using BioEdit software [20] and OligoCalc (http://biotools.nubic.northwestern.edu/OligoCalc.html) (Table-1) [21]. The real-time RT-PCR reaction mixture was prepared in a total volume of 10 μL. The mixture consisted of 5 μL of SsoAdvanced Universal SYBR Green Supermix (Bio-Rad, USA.), 0.25 μL of each primer (Table-1) [21], 2.5 μL of nuclease-free water, and 2 μL cDNA. The amplification cycles were conducted with an initial denaturation step at 95°C for 3 min, followed by 40 cycles of denaturation at 95°C for 30 s, annealing at 55°C for 30 s, and extension 72°C for 30 s. The positive control for the amplification reactions included the JEV RNA Nakayama GIII strain and a clinical strain of ZIKV provided by the Armed Forces Research Institute of Medical Sciences (AFRIMS, Thailand). A reaction mix without a viral cDNA template was used as a negative control. Real-time RT-PCR products of positive or suspected pools were collected and diluted at ratios of 1:10 and 1:100 to confirm the presence of both viruses detected in these samples by nested real-time PCR using primers (Table-1) [21]. The results were analyzed using melting temperature (Tm) and threshold cycle (Ct) values for the amplification plots for virus identification. PCR products were then visualized by gel electrophoresis.

**Table-1:** Primers used in real-time RT-PCR amplification.

Virus	Target genes	Primer sequence (5’- 3’)	Reference
JEV	E and partial NS1	F: GGTGCATTCAGAACRCTCTTYG R: GTGTCRGCATGCACATTGGTMGC	-
ZIKV	E	F: AGYCGYTGYCCAACACAAG R: CACCARRCTCCCYTTGCCA	[21]

RT-PCR=Reverse transcription-polymerase chain reaction, JEV=Japanese encephalitis virus, ZIKV=Zika virus

### Meteorological data collection

The Thai Meteorological Department provided the meteorological data for both sample sites. Monthly data of rainfall (mm), minimum temperature (°C), and maximum temperature (°C) were collected from the Northeastern Meteorological Center (Lower Part), Ubon Ratchathani province.

### Statistical analysis

The statistical analyses were conducted with Microsoft Excel (Office 365) version 16.78.3 (Microsoft Corp., Washington, USA). The mean numbers of *Culex* and *Aedes* mosquitoes were calculated across all study sites in Mueang and Det Udom districts. A two-tailed t-test was used to examine the disparity in mean populations of *Culex* and *Aedes* mosquitoes at each study site. The relationship between the monthly abundance of *Culex* and *Aedes* mosquitoes (number) and monthly rainfall (millimeter) was analyzed at the sample sites. Statistical analyses were carried out with p < 0.05 as the threshold for significance.

## Results

### Distribution of *Culex* mosquitoes and weather information

**Figure-1:**
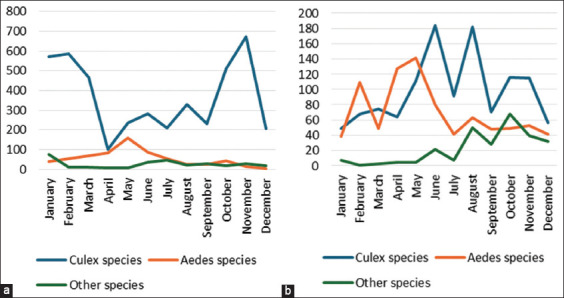
The number of female *Culex*, female *Aedes*, and other mosquito species collected from study sites in 2019. (a) Number of mosquitoes collected from Don Yung, Mueang district, and (b) Number of mosquitoes collected from Phon Duan, Det Udom district, respectively.

Sample pools (335) contained a combined total of 5587 adult female *Culex* mosquitoes collected from Ubon Ratchathani. The collected samples from Don Yung, Mueang district were 4,406 (78.9%), while from Phon Duan, Det Udom district were 1,181 (21.1%). Other mosquito species, including *Aedes*, were also identified. In Don Yung, *Culex* mosquitoes comprised over 80%, while in Phon Duan, they accounted for 50% of the total mosquito catch (Table-2). In Phon Duan, there were more *Aedes* mosquitoes than in Don Yung. In Don Yung, the *Culex* species outnumbered *Aedes* species (p < 0.05), but there was no significant difference in mosquito numbers between Phon Duan and the other location (p > 0.05). In November, Don Yung recorded the highest mosquito population (674) and Phon Duan peaked in June with 184 mosquitoes (Figure-1). *Aedes* mosquito numbers peaked in May for both districts (Figure-1).

**Table-2:** Overview of mosquito genera collected from sample collection sites in Ubon Ratchathani during January–December 2019.

Sample collection site and mosquito species	Don Yung, Mueang district			Total	Phon Duan, Det Udom district			Total
	
	*Culex* species^a^	*Aedes* species^b^	Other species		*Culex* species^a^	*Aedes* species^b^	Other species	
Number of mosquitoes collected during 2019	4,406	662	312	5,380	1,181	839	264	2,284
Percentage	81.9	12.3	5.8	100	51.7	36.7	11.6	100
The average of mosquito number	367.2*	55.2*	26	-	98.4**	69.9**	22	-

a Total of female of *Culex* spp. was represented.

b Total of female *Aedes aegypti* and *Aedes albopictus* mosquitoes.

* *Culex* number was showed higher than *Aedes* number with significantly (p < 0.05).

** Number of *Culex* and *Aedes* species collected from Phon Duan have no difference (p > 0.05)

**Figure-2:**
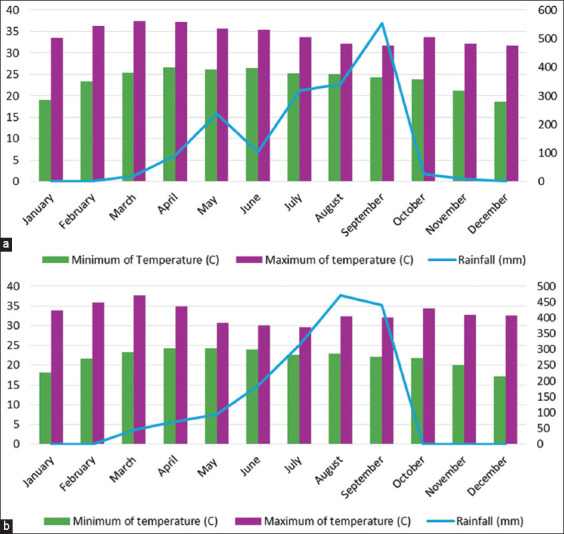
Meteorological data of study area; (a) Mueang district and (b) Det Udom district.

According to the Thai Meteorological Department, Mueang district had average temperatures ranging between 23.7°C and 34.2°C and an annual rainfall of 1698 mm, while Det Udom district had temperatures from 21.8°C to 33.1°C and an annual rainfall of 1621 mm (Table-3). In Mueang and Det Udom districts, the highest rainfall was recorded in September and August, respectively (Figure-2). This study found no significant correlation between monthly rainfall and the populations of *Culex* (r = −0.18) and *Aedes* (r = −0.04) mosquitoes across the study sites (p > 0.05).

**Table-3:** The minimum or maximum temperatures and monthly rainfall in Mueang and Det Udom district in 2019 reported by Thai Meteorological Department.

Months	Mueang district			Det Udom district		
	
	Temperature (°C)		Rainfall (mm)	Temperature (°C)		Rainfall (mm)
	
	Min	Max		Min	Max	
January	19.1	33.5	0.0	18.2	33.8	0.0
February	23.4	36.3	0.0	21.6	35.8	0.0
March	25.3	37.3	19.5	23.3	37.6	45.6
April	26.6	37.2	91.4	24.3	34.8	71.7
May	26.1	35.6	236.3	24.2	30.7	94.3
June	26.4	35.3	102.0	23.9	30.1	184.9
July	25.2	33.7	318.5	22.7	29.6	313.4
August	25.0	32.1	341.9	22.9	32.4	471.5
September	24.3	31.7	554.5	22.2	32.1	439.6
October	23.8	33.6	26.6	21.8	34.4	0.0
November	21.2	32.1	7.0	19.9	32.7	0.0
December	18.5	31.7	0.0	17.1	32.5	0.0
Mean	23.7	34.2	141.48	21.8	33.1	135.08

### JEV and ZIKV detection in mosquitoes

To determine mosquito RNA quality, 86 RNA pools, representing 25.7% of the sample (86/335), were subjected to randomization and amplification of the mosquito actin-1 gene by real-time RT-PCR. The Tm value of actin-1 from mosquito pool samples displayed multiple peaks, including a temperature range of 83.1°C–84.3°C and 88.0°C–90.0°C with distinct Ct. Forty tested samples showed positive at two peaks, including the range of 83.1°C–84.2°C and 88.0°C–90.0°C, and 14 and 32 sample pools were found at one peak at the range of 83.8°C–84.3°C and 88.0°C–89.9°C, respectively (Table-4).

**Table-4:** Number of mosquito pool samples represented with different Tm.

Temperature range	Number of mosquito pool samples (n = 86) (%)
83.1°C–84.2°C and 88.0°C–90.0°C (2 peaks)	40 (46.5)
83.8°C–84.3°C (1 peak)	14 (16.3)
88.0°C–89.9°C (1 peak)	32 (37.2)

Tm=Melting temperature

**Figure-3:**
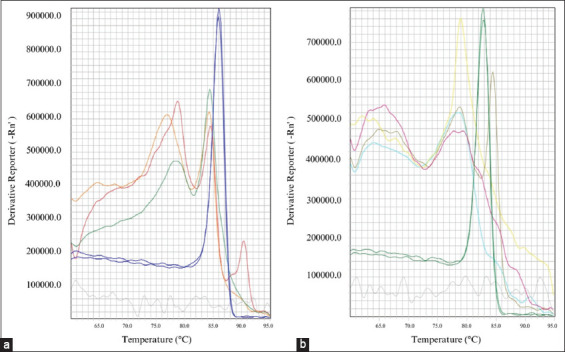
The melting temperature (Tm) of positive controls compared with Tm value of tested samples; (a) Japanese encephalitis virus (JEV) Nakayama GIII strain, a positive control, showed Tm value of 86.0°C (blue line) and a green, red, and orange lines were the Tm value of the mosquito samples detected by Japanese encephalitis specific primer. (b) Zika virus (ZIKV) positive control was presented as dark green line with Tm value of 82.7°C and yellow, pink, turquoise blue, and olive-green lines were the Tm value of mosquito samples detected by ZIKV specific primer. The gray lines in these figures are the Tm value of negative control.

RNA pools’ viral cDNAs (335) were analyzed using real-time RT-PCR to determine the presence of JEV and ZIKV in *Culex* mosquitoes. The JEV Nakayama GIII strain’s Tm value, serving as the reaction’s positive control, was 86.0°C. Figure-3 indicates a Tm value of 82.7°C for the ZIKV product. Figure-3 shows no identical Tm peak was found in any viral cDNA pool from field-caught female adult *Culex* mosquitoes compared with the positive control. All tested samples were found to be negative for JEV and ZIKV.

## Discussion

Mosquito-borne diseases such as dengue, Japanese encephalitis, Zika fever, malaria, and filariasis pose significant public health issues in Thailand. Although nationwide vaccination campaigns against JE have significantly reduced the number of cases, sporadic JEV-infected cases occur annually in Thailand, including in the Ubon Ratchathani province until 2021 [4]. In 2016, it was reported in Thailand that *Culex* mosquitoes contribute to ZIKV transmission [9, 14]. We performed 1-year molecular surveillance of JEV and ZIKV in female adult mosquitoes from the urban Mueang district and rural Det Udom district within Ubon Ratchathani province.

In both study areas, *Culex* species was the most abundant mosquito species, as shown in other studies employing a range of trapping techniques in Thailand [22–26]. Nitatpattana *et al*. [5] collected JEV-positive *Culex* mosquitoes using CDC gravid traps (Model 1712) and CDC light traps in 2003 during a JEV distribution study. We used a Prokopack aspirator for sample collection in our standard method, but not in that case. The study by Vazquez-Prokopec *et al*. [18] demonstrated that the Prokopack aspirator can effectively collect different mosquito species compared with the CDC Backpack Aspirator (CDC-BP) [18]. *Culex* mosquito species were not identified in this study. Following morphological identification, we grouped mosquito genera using the collected samples. Despite long-term storage, some identifying details of the mosquito species in this study were lost due to the complete decomposition of major body parts. Identification of *Culex* species is challenging due to these reasons. Quantitative PCR molecular techniques are recommended for mosquito species identification, providing valuable insights for studying and analyzing mosquito distributions.

In Thailand, mosquito distribution typically increases during the rainy season (May–October) and declines during the cold and hot seasons (January–April) [26, 27]. During the rainy season, Aedes are commonly found. However, the pattern of distribution was not found in *Culex*, similar to other studies, which presented the highest and lowest *Culex* numbers in November (transitional season) and September (rainy season), respectively [26]. The study revealed a higher prevalence of *Culex* mosquitoes in urban environments. These findings revealed that the relationship between mosquito distribution, human habitats, and environmental change, which affect mosquito vectors, may increase the risk of re-emerging infectious diseases and human health in the future [25].

Understanding the epidemiology of JEV and ZIKV in *Culex* mosquitoes, even if not detected as infected in our study, could shift the prevention approach. Multiple studies from different areas in Thailand have reported negative JEV detection in collected mosquito genera, apart from our study. Tiawsirisup *et al*. [23] and Tiawsirisup and Nuchprayoon [26] reported no detection of JEV in mosquitoes from migratory birdnesting areas in Pathum Thani province from March 2008 to January 2009, or in bats and their habitats in Lopburi province from May 2009 to April 2010 using RT-PCR technique. Mosquitoes from the ardeid birds’ nesting colony in Phitsanulok province did not detect JEV [22].

According to Olsen *et al*. [2], 15% of reported acute encephalitis syndrome cases were due to JEV infection. In Thailand, hospitalized patients continue to contract encephalitis from JEV. Eight cases of JE were reported to the Bureau of Epidemiology, MoPH in 2019 [4]. The majority, 87.5% (7/8 cases) of JEV cases were found in the Ubon Ratchathani province, particularly in the Khemarat (N16.0417778, E105.2064444), Na Yia (N15.2926667, E104.8203056), Warin Chamrap (N15.1941111, E104.8381667), and Samrong (N15.0278611, E104.7743611) districts. In Mueang (N15.2926667, E104.8203056) and Det Udom (N14.6471944, E105.0929167) districts, despite being neighbors of previously JEV-endemic regions, no JEV was detected.

Recent research indicates that *Culex* mosquitoes play a significant role in transmitting ZIKV. Mosquito genera containing ZIKV at various developmental stages were detected through RNA detection. About 1.85% of *Cx. quinquefasciatus* females, 1.66% of males, and 0.29% of larvae tested positive for ZIKV. About 2.24% of women, 1.27% of male *Aedes* adults, and 0.19% of *Ae. aegypti* larvae were ZIKV positive, according to Phumee *et al*. [9], whereas no female adult *Culex* mosquitoes were positive. About 0.73% of hospitalized patients in Ubon Ratchathani province were diagnosed with ZIKV.

## Conclusion

The present study failed to detect JEV and ZIKV in female adult *Culex* mosquitoes from Ubon Ratchathani province. This may be attributed to the low viral load circulating within the vectors in the area, rendering detection challenging, or it may be because *Culex* mosquitoes are not suitable vectors for the virus under investigation. During interepidemic periods, this information reveals the secret transmission of arboviruses by their theoretical vectors. Studies on mosquito populations, asymptomatic patients, and reservoir hosts such as pigs and water birds, particularly in epidemic areas with larger sample sizes, remain essential for advancing our understanding of JEV transmission dynamics. In the epidemic area, comprehending the patterns of arboviral circulation is crucial for preparing for future outbreaks.

## Authors’ Contributions

WS, CP, TE, HJO, SA, and SP: Conceptualization and methodology. WS and SP: Data curation and formal analysis and validation. WS: Writing the original draft. SP, CP, TE, and HJO: Writing, review, and editing of the manuscript. SP: Supervision. CP, TE, and SP: Investigation, HJO: Project administration. CP, TE, and HJO: Funding acquisition. All authors have read, reviewed, and approved the final manuscript.
